# Effectiveness of an intervention for prevention and treatment of burnout in primary health care professionals

**DOI:** 10.1186/1471-2296-14-173

**Published:** 2013-11-17

**Authors:** Tomás Gómez-Gascón, Jesús Martín-Fernández, Macarena Gálvez-Herrer, Ester Tapias-Merino, Milagros Beamud-Lagos, José Carlos Mingote-Adán

**Affiliations:** 1 Guayaba Health Center, Primary Care, Madrid Health Service, Madrid, Spain; 2 Villamanta Health Center, Primary Care, Madrid Health Service, Madrid, Spain; 3 PAIPSE (Programa de Atención Integral al Profesional Sanitario Enfermo), Madrid Health Service, Madrid, Spain; 4 Comillas Health Center, Primary Care, Madrid Health Service, Madrid, Spain; 5 El Greco Health Center, Primary Care, Madrid Health Service, Madrid, Spain

## Abstract

**Background:**

Burnout syndrome is an important health problem that affects many professionals and must be addressed globally, with both organizational measures and personal interventions. Burnout of health professionals can be prevented in order to avoid personal, familial, and social consequences, as well as repercussions for patients.

**Methods/design:**

This work describes a protocol for a controlled, pragmatic, randomized clinical trial in 2 parallel groups: intervention and control. All health professionals from 7 health care centers will form the intervention group, and all health professionals from 7 different health care centers will form the control group. The intervention group will receive 16 hours of training at their work place. The Maslach's burnout inventory, the Cuestionario de Desgaste Profesional Médico or the Cuestionario de Desgaste Profesional de Enfermería, and the 28-item Goldberg's General Health Questionnaire, validated for our setting, will be used as measurement tools. Change in the average scores from the Maslach's burnout inventory emotional exhaustion scale will be compared between the intervention and control groups, measured as intention-to-treat, and the intervention will be considered effective if a minimum decrease of 20% is achieved.

**Discussion:**

Due to the deleterious consequences of burnout syndrome for people suffering from it and for the organization where they work, it is necessary to evaluate the effectiveness of certain interventions for its prevention. Organizational measures are important for preventing burnout syndrome, but so is providing professionals with coping strategies, as this group intervention intends to do.

**Trial registration:**

ClinicalTrials.gov processed this record on June 10, 2013. ClinicalTrials.gov Identifier: NCT01870154.

## Background

There is a high prevalence of job burnout or profession related wearing down, which is considered to be an adaptive disorder to chronic work stress and entails harmful consequences for the individual suffering from it and the employing organization [1].

Studies performed in primary care show that around 40% of family doctors suffer from burnout syndrome to some degree [2], although with variable results ranging from 10% to 80% [3].

Concern about the satisfaction of health professionals led to initial studies by Donabedian in 1966, and later by Freebon and Greenlick in 1973.

Donabedian states that the degree of quality of the services provided by a health care system is directly related to the satisfaction level of the professionals working within it, and reveals that their demoralization is the main difficulty faced by directors and managers of health care centers (HCCs).

Work motivation is the result of a series of interactions between individual effort, obtained output, organizational rewards, and personal objectives. Even though any worker is susceptible to dejection, those most likely to suffer from it are the ones directly in contact with the public.

The term “work stress” was first introduced by McGrath in 1970 and defined as the imbalance perceived between a demand and the individual's capacity to fulfill it under conditions where failing to fulfill that demand entails significant consequences.

Even though Donabedian and Freebon were the first ones to associate work satisfaction with the quality of supplied services, it was not until 1974 that psychoanalyst Herbert Freudenberger talks of “work disease” for the first time, defining the “burnout syndrome” as a state of exhaustion or frustration resulting from dedication to a cause, way of life, or relationship that does not result in the expected reinforcement [4].

Herbert Freudenberger mentions that this syndrome is more frequent in helping professions, where facing intense emotions of pain, disease, and/or psychological suffering is common.

Health professionals work with the most intense emotional facets of human beings (suffering, fear, sexuality, and death), and they cannot, nor must, be completely indifferent towards them. Thus, it is easy for them to be subjected to a high degree of chronic stress and a state of physical and psychological exhaustion.

Stress is the response (physiological, psychological, and behavioral) of beings when facing events, situations, people, or objects perceived as stressful, which consequently induces a stress response that is essential for survival. According to Hans Seyle, the 3 physiological phases that the response follows are: alarm, adaptation or resistance, and exhaustion. During stressful situations, attention increases and the brain focuses on the perceived challenge. When stress becomes chronic, it stops being a physiological stimulus and becomes detrimental to health [5].

In 1982, psychologist Maslach describes the responses of workers to different stressful situations in their work life, and tries to define the basic characteristics of the syndrome that she terms “burnout syndrome” [6].

The basic characteristics of the syndrome are: emotional exhaustion, feeling of fatigue with both mental (anxiety, anguish, sexual dysfunction, chronic fatigue, etc.) and physical (spastic colon, dyspepsia, headache, myalgia, etc.) manifestations, and depersonalization. Depersonalization results in isolation behaviors, insensitivity, dehumanization, negativity, distancing from patients, low personal fulfillment (dissatisfaction about professional achievements and a desire to give up). In the health setting, mental fatigue results in a state of exhaustion in the face of those task requirements that the worker feels no interest to perform; depersonalization implies a rejection behavior towards patients, referring to them as objects; and lack of personal fulfillment consists of a negative attitude towards oneself and work, loss of interest towards work, irritability, low productivity, and low self-esteem. Thus, the burnout syndrome has important family and social repercussions, as well as for the work environment and organization, which translate into absenteeism from work, decrease of worker and user satisfaction, job mobility, and loss of productivity [3,5,7].

In the health setting, there is a serious concern about the quality of the care provided to the community and user's degree of satisfaction, whereas less attention is dedicated to the work-related health of the professionals. However, it has been demonstrated that dissatisfaction of health professionals entails an important economic and social cost due to its effect on the work environment, performance, and supply of health care.

For the last decades, we are experiencing a gradual increase of health care demand as a consequence of a population that is more critical and demanding of the care provided by health professionals. Additionally, this modification of health habits in the general population has not been accompanied by the administrative and managerial strategies necessary to provide an appropriate health care work environment.

Work satisfaction has not significantly changed in the last 10 years despite the important changes introduced by the reform of the Spanish primary care system. There is evidence that some aspects related to work satisfaction (work excess, labor tension, and promotion) have worsened. These factors can be a source of stress that leads to burnout when they are permanently maintained and overcome the individual's self-defense mechanisms. Variables related to the syndrome are excessive number of patient consultations, excessive responsibility over clinical decisions that exceed the level appropriate for the primary care setting, and difficulties in accessing continuing medical training [8].

In environments that are prone to generating this disorder, a series of factors are usually observed at an organizational level: organizational bureaucratic culture with scarce actual participation in the decision-making processes that may affect the staff; performance evaluation based on quantitative aspects and limited appreciation of qualitative ones; poor organization of tasks, job assignments, and macro-social factors such as degree of complexity and difficulty, lack of supervisor and partner support, scarce encouragement for team work, unjustified lack of resources, low or no planning, few possibilities for promotion; and additional factors [9].

Training programs aimed at preventing burnout syndrome must include training at various levels. At an organizational level, training workers in organizational development and change is required. The inter-personal level takes into account working groups and social interaction, and therefore training programs on social support, social skills, self-efficacy, and leadership, among others, must be included. The individual level must provide solutions to personal needs for coping with stress [10].

Since stress is unavoidable, it is most important to educate the worker on how to deal with it as efficiently as possible [11,12]. It is estimated that around two thirds of all diseases are related to stress, and even though it is not a cause-and-effect relationship, stress is an important factor that interacts with other biological, psychological, and social variables, resulting in numerous physical and mental pathologies [13].

Stress coping techniques aim to modify any aspects of the triple response (psycho-physiological, cognitive, and behavioral) generated by the organism when facing stress [14].

Control over activation of the psycho-physiological component allows the person to cope with daily life stressors by controlling physical symptoms resulting from the generated anxiety (palpitations, dizziness, muscle tension, etc.).

Techniques on controlling the initiation of the response are easily learned strategies that do not require much training time and can be acquired at any age. They can be carried out wherever the individual is, without the need for special conditions for their application. With adequate learning, they are able to control stress and anxiety almost immediately [15].

People are the most important resource in the health care system for achieving its objectives. The burnout syndrome is considered to be an adaptive disorder to chronic work stress [1], therefore stress-interventions for health professionals will contribute to reducing it and consequently improve population care.

In a previous research project developed by our group, *Effectiveness of two interventions on primary health professionals with burnout for dealing with stress*, of which 2 partial articles have been published [16,17], it was proven that burnout is a very wide-spread problem and carrying out a trans-personal relaxation workshop improves psycho-pathologies, as measured by Goldberg's test, and emotional exhaustion, the most important component of burnout, as measured by the Maslach's burnout inventory (MBI).

From a clinical perspective, burnout can be considered a type of adaptive disorder related to psycho-social work stressors and the existence of a persistent imbalance between demands and available resources for fulfilling them, expectations and perceived results, as well as performed effort and obtained reinforcements. The pathology results from an accumulation of risk factors, whether they are personal, organizational, work-related, or other, and from the lack of protective factors and resistance to experienced stress [3].

Apart from the need for organizational measures to improve the situation, establishing interventions aimed at minimizing the prevalence of burnout syndrome may be useful. Thus, we consider this study as a continuation to the previous one, seeking an effective intervention aimed at all health professionals within a primary care team.

The novelty of this study is that the intervention will be performed on all health professionals from a primary care team, acting on both personal and inter-personal levels, which we estimate will reinforce the effect of the intervention. Some of the main variables that affect organizational behavior, and therefore the appearance of the burnout syndrome, are found in the social-interpersonal realm. This syndrome has its origin in the interpersonal professional relationships that are established within the work environment. The objective is to train workers to improve social processes (formal and informal) that are developed during their work activities [10].

## Methods/design

### Design

Controlled, pragmatic, randomized clinical trial with an intervention and control group that will not present differences in any studied characteristics *a priori*.

All medium to large size HCCs from the area will be offered participation and randomly allocated, 7 to the intervention group and other 7 to the control group.

### Studied subjects

The studied population is comprised of all health professionals from 14 primary health care teams of in Area 11 of Madrid who accept to participate (a total of 400 subjects are anticipated to be included).

Inclusion criteria: All health professionals (family physicians, pediatricians, and nurses) who are part of the health care centers (HCC) at the moment of the trial.

### Sample size and sampling

The main outcome variable will be the score on the emotional exhaustion sub-scale of the MBI.

In a previous study performed by this group in the same setting, the average score on this sub-scale was 24, with a standard deviation of 12 points. A change of at least 5 points with respect to the baseline (~20% of the initial value) will be considered significant. These figures do not differ from those found in other populations.

The sample is calculated as non-finite size data, using the formula proposed by Snedecor and Cochran [18].

It is therefore necessary to include ~90 subjects per group, considering a type I error <0.05 and a type II error of 20% (statistical power 80%).

However, we are going to study clusters (health centers) where the variability of the studied parameter will be lower among subjects from the same group and higher among subjects from different groups. This effect, termed the “design effect”, multiplies the needed sample size by a factor ranging from 1.5 to 5. The magnitude of this factor depends on the relationship between the intragroup and intergroup variance, termed the intraclass correlation coefficient [19]. In this case, since there are environmental factors that favor the appearance of burnout, at least moderate intraclass clustering is expected, for which we estimate the design factor will be ~2. Thus, we will need at least 180 subjects per group. If we have a loss to follow-up of ~10%, the studied population will have to be ~200 subjects per group.

### Intervention

The intervention consists of 16 hours of training, to be held at the subject’s HCC. The workshop involves mixed learning, comprising 4 sessions, each 2 hours long, in addition to personal work previous to and after each session of reading relevant bibliography and performing exercises, self-evaluation, and case studies (8 hours of individual work).

The objective of the intervention is learning from the work experience of professionals attending the workshop to know and recognize the risk and process of the burnout syndrome and the characteristics specific to the health setting, as well as promoting prevention lines by transmitting strategies to manage and control the elements and consequences of the syndrome process (physical, psychological, and social).

Session 1. The burnout syndrome: identifying and coping with the main job stressors.

Session 2. The process of burnout: cognitive and motivational components.

Session 3. Moderating factors of the burnout process: emotional competence and positive personality.

Session 4. Managing the consequences of the burnout: self-care and recovery.

### Randomization

The HCCs that accept to participate will be grouped in pairs based on 3 features: number of professionals, socio-economic area, and urban or rural environment. One center from each pair will be randomly allocated into intervention or control group, and the paired center into the opposite group (allocation by blocks of size 2).

### Measurement tools and included variables

#### Measurement tools

Worker classification regarding burnout syndrome will be performed according to the MBI, which includes 22 items to evaluate emotional exhaustion (EE), depersonalization (DP), and personal fulfillment (PF), with scores ranging from 0 to 6 on the Likert scale. We consider a high degree of burnout in the case of EE ≥ 27 points, DP ≥ 10, and PF <33. Moderate burnout will be considered in the case of 26 < EE < 19 points, 6 < DP < 9 points, and 34 < PF < 39 points. Low levels will be considered for EE ≤ 18 points, DP ≤ 5 points, and PF ≥ 40 points.

Additionally, 2 specific questionnaires for measuring burnout syndrome will be used: the Cuestionario de Desgaste Profesional Médico (CDPM) and the Cuestionario de Desgaste Profesional de Enfermería (CDPE), validated for out setting [3,20].

The CDPE comprises 65 items, with scores ranging from 1 to 4, and evaluates work stressors, professional burnout, resistant personality, and coping.

The CDPM comprises 4 blocks: antecedent variables scale with 24 items, scale of medical burnout syndrome with 12 items, consequent variables scale with 16 items, and scale of positive personal resources with 16 items. Each item is given a value from 1 to 4.

The evaluation of psycho-pathological characteristics of subjects will be carried out using Golberg's GHQ, validated in Spanish by Lobo et al. in 1985 [21]. It consists of 28 items, divided into 4 sub-scales. It is used to estimate the prevalence of psychiatric disease in a specific population and discover cases of psychiatric pathologies in non-specialized consultations. It is not appropriate for performing clinical diagnosis, but can be employed to evaluate symptomatology using its sub-scales: A (somatic symptoms), B (anxiety/insomnia), C (social dysfunction), and D (severe depression). There are 4 possible answers to each question. The Likert scoring system assigns a value from 0 to 3 to each of the 4 possible answers.

### Studied variables

#### Independent variable

Group intervention consisting of 16 hours of training, to be held at the health professional’s own HCC.

#### Dependent variable

Possible burnout condition as identified by score modification, or change, before and after the intervention on the EE scale of the MBI for each subject and for the whole sample.

#### Control variables

● Demographic: age, sex, marital status, number of children, and years of cohabitation with couple.

● Professional category: family doctor, pediatrician, or nurse.

● Years of experience in primary care.

● Type of contract: interim or permanent.

● Performance of tasks involving responsibility (coordination, group leader, supervisor).

● Average practitioner workload per working day.

● Working in shifts.

● Work leave >1 month in the last 6 months.

● General health condition, measured with Goldberg's GHQ-28.

### Data analysis

#### Descriptive statistics

A study on prevalence of “job burnout” in the population will be performed, as well as on their socio-demographic characteristics and work conditions.

Quantitative variables will be described according to their measures of central tendency, mean, and confidence intervals in the case of normal distributions, and by their median and interquartile range in the case of non-normal distributions.

Percentages and confidence intervals will be obtained for qualitative variables.

#### Analytical statistics

Initial comparability between the intervention and control groups will be analyzed with the Student's t-test in the case of independent data of quantitative variables (age, years of experience, workload, scores from the questionnaires, etc.) that fit a normal distribution, with the Mann-Whitney U non-parametric test for those not fitting a normal distribution, and with contingency tables in the case of qualitative variables (sex, marital status, professional group, type of contract, etc.).

Univariate correlations between socio-demographic and work characteristics of subjects and their score in the MBI at the beginning of the trial, as well as the correlation between the mentioned characteristics and the change observed during the follow-up, will be analyzed by use of simple linear regression models.

The correlation between the changes associated with burnout syndrome and changes in work-life quality and anxiety, as perceived by health professionals, will be measured using the correlation coefficients for score changes from the MBI, the professional quality of life questionnaire (QPL-35), and Goldberg's GHQ. Pearson's r will be used in the case of normal distributions, and Spearman's rho for non-normal distributions.

Explanatory models will be built to identify work and occupational characteristics related to work satisfaction and burnout. The dependent variable will be the score obtained from the relevant questionnaires at baseline, and the independent variables those considered as relevant in previous theoretical models or proven significant in the univariate analysis.

A comparison of average scores on the MBI will be performed between the intervention and control groups after the intervention and at the end of the follow-up in order to measure the effectiveness of the intervention, and the Student's t-test will be employed for independent data. Confidence intervals of 95% will be calculated for the differences. If the application criteria are not met for that analysis, non-parametric tests will be used (Mann-Whitney U test).

An explanatory model will be constructed to provide an answer to the principal objective, the dependent variable will be score on the MBI, the main independent variable will be the intervention, and modifying variables will be all those found to be related to burnout in the univariate analysis or the signficant ones in theoretical models. This analysis will complement the first one since it will allow for correcting for confounding factors and analyzing effect modifications.

Since subjects belong to various clusters (health centers), generalized estimating equations (GEE) models will be used to build explanatory models that serve for identifying work and occupational characteristics related to work satisfaction and burnout, or to provide an answer to the principal objective. GEE models correct for non-independence of observations among the same group (health center), assuming *a priori* a certain structure of correlation for the dependent variable measured in each group. In addition, these models are not very stringent about the distribution of the outcome variable and offer standard error measurements of the “robust” coefficients, which are stable even if the correlation chosen *a priori* is incorrect or if the strength of the correlation between the observation changes along the different clusters [22]. In their interpretation, however, they do not allow measuring the influence that a characteristic has on each subject, but they allow for estimating the average of the population response when the independent variables change for the whole population [23].

#### Ethical considerations

This study has been approved by the Clinical Research Ethics Committee of Hospital Doce de Octubre in Madrid. It has been registered under reference code 09/094.

The whole research process will be governed by the ethical principles contained in the Declaration of Helsinki (revision Seoul 2008). Included subjects will be asked for their written consent to participate in the study and informed that all data will be kept and treated anonymously in compliance with the requirements of national legislation.

## Discussion

Burnout syndrome is an important health problem that affects many professionals and must be globally addressed with organizational measures and personal-level interventions.

In this work, a continuation of a previous project termed “*Effectiveness of two interventions on primary health professionals with burnout for dealing with stress*”, we will evaluate a group intervention for preventing burnout, consisting of a training to be held at the workers' own HCC.

This intervention has been chosen due to its feasibility and reproducibility, because it does not require many resources, and because can be carried out in all HCCs where it may be needed. Since we are dealing with a major problem, we seek a tool that can be used by many professionals in the future. A more prolonged intervention would have been preferable in order to obtain a more favorable outcome, but would not be translatable for the current work reality of Spanish HCCs.

Contrary to our previous work, the intervention is performed at the worker's own HCC in order to contribute a component that improves the work place environment and team work.

There is much to be researched on this complex syndrome that is becoming more frequent, jeopardizes the health of many professionals, and secondarily affects the care of patients.

The current economic crisis is reducing health resources and at the same time increasing psycho-social problems of patients, and therefore a rise in the prevalence of burnout syndrome is foreseeable.

The MBI questionnaire is used in this project since it is the most frequently employed tool for assessing this syndrome, has contributed the most to its study, and acceptable values are obtained from the point of view of validity [24]. However, it is not free of psychometric problems, showing some weakness, especially outside Anglo-Saxon regions [25].

Therefore, two other questionnaires have also been included: a doctor specific one, the CDPM, and another for nurses, the CDPE. Studies based on the MBI have mainly focused on the dimensionality of the construct (the burnout syndrome), whereas less attention has been paid to the elements of the process: antecedent and consequent variables, as well as personal modulators. The CDPM and the CDPE focus primarily around the different phases of the process: antecedent variables, syndrome expression, outcomes or consequences, and personal positive resources. Variables of positive personality are associated with the various elements of the process of burnout.

The work of health professions is highly stressful and entails a risk for burnout. Complete elimination of some of its triggers is practically impossible in many occasions. However, the effect of these stressors can be moderated by personal resources. In view of this, empowerment and development of these resources among health professionals will be an important preventive measure to fight professional burnout through secondary prevention [3].

Whether burnout syndrome can be nosologic is debatable [26], but it is clear that it entails severe physical, mental, and social consequences. Regardless, it is important to act and provide people with strategies and effective tools to be able to adapt to those situations that are perceived as highly stressful [27].

## Competing interests

None of the authors has any economic conflict of interest. All the authors, except VLA, work in the public health system.

## Authors’ contributions

TGG conceived and participated in the design of the study, draft the manuscript, and reviewed the final manuscript. JMF, MGH participated in the design of the study and reviewed the final manuscript. MBL and JCMA participated in the design of the sutdy. ETM reviewed the final manuscript. The members of EDESPROAP-Madrid Group and ETM contributed to the implementation of the study. All authors read and approved the final manuscript.

## Pre-publication history

The pre-publication history for this paper can be accessed here:

http://www.biomedcentral.com/1471-2296/14/173/prepub

## Grupo EDESPROAP-Madrid (Grupo de Estudio del Desgaste Profesional en Atención Primaria de Madrid)

Francisco Javier Peces Morate; Francisco López López; Carlos Martínez García-Olalla; Javier Vaquero Argüelles; Vanesa Lara López Agrelo, Legal and Clinical Psychologist in Forensic Psychology Unit of the Lopez-Ibor Clínic; María Medina Sampedro, Villaviciosa de Odón Health Center; Julia Caballer Rodilla, Silvia Durán Francisco, El Restón Health Center; Mª del Pilar López Morandeira, Sonia Rúa Sanz, Aquitania Health Center; Juan Carlos Muñoz García, Móstoles El Soto Health Center; Concepción Aragoneses Isabel, Lucero Health Center; Luisa María Postigo Higuera, Fabiola Arbocco Morales, Dos de Mayo Health Center, Móstoles; Esteban González López, Villanueva de la Cañada Heatlh Center; Javier Bris Pertíñez, Rafael Joaquín García Delgado, Villa de Vallecas Health Center; Raúl Sánchez González, Mercedes Figueroa Martín-Buitrago San Blas Health Center, Parla; Beatriz López Uriarte, Francisco Góngora Maldonado, Humanes Health Center; Tomás Gómez Gascón, Mª Angeles Gómez Medina, Paloma Aguilera Reija, Guayaba Health Center; Rosario Ureña Durán, Marta Martínez Gómez, Caramuel Health Center; Elena Martín Ramos, Alcalde Bartolomé González Health Center, Móstoles: Miguel Angel Delgado Nicolás, Francisco Javier Amador Romero, Los Angeles Health Center; Ester Tapias Merino, Comillas Health Center; Diego Martín Acicoya, Calesas Health Center.
